# Does fixed retention prevent overeruption of unopposed mandibular second molars in maxillary first molar extraction cases?

**DOI:** 10.1186/s40510-016-0119-z

**Published:** 2016-01-21

**Authors:** Christos Livas, Demetrios J. Halazonetis, Johan W. Booij, Christos Katsaros, Yijin Ren

**Affiliations:** Department of Orthodontics, University of Groningen, University Medical Center Groningen, Hanzeplein 1, Triade gebouw, Ingang 24, 9700 RB Groningen, The Netherlands; Department of Orthodontics, School of Dentistry, National and Kapodistrian University of Athens, 2 Thivon Str, 115 27 Goudi, Athens Greece; Private practice, Schelluinsevliet 5, 4203 NB Gorinchem, The Netherlands; Department of Orthodontics and Dentofacial Orthopedics, School of Dental Medicine, University of Bern, Freiburgstrasse 7, CH-3010 Bern, Switzerland

**Keywords:** Tooth overeruption, Fixed retainers, Unopposed molars, Maxillary first molar extraction

## Abstract

**Background:**

The objective of this study was to investigate whether multistranded fixed retainers prevented overeruption of unopposed mandibular second molars in maxillary first molar extraction cases.

**Methods:**

The panoramic radiographs of 65 Class II Division 1 Caucasian Whites (28 females, 37 males) consecutively treated with bilateral maxillary first molar extraction and the Begg technique, and with records taken after treatment (T1) and in retention (T2), were withdrawn from private practice records. After appliance removal, mandibular second molars were retained with sectional wires till at least T2 in case of lack of occlusal contact with the antagonist. The subjects were assigned to study-retention and control-nonretention groups based on the retention status of mandibular second molars. Radiographic analysis was carried out to determine inclination of mandibular molars and the resulting movement of second molar centroids. Parametric and nonparametric tests were performed to assess the changes between T1 and T2.

**Results:**

No statistically significant differences in molar inclination were observed between groups and timepoints (*P* > 0.05). There were no statistically significant differences in molar movement percentages (*P* > 0.05) irrespective of whether fixed retention had been used or not.

**Conclusions:**

No significant eruption occurred in unopposed mandibular second molars bonded with fixed sectional retainers compared to molars partially occluded with the antagonists without fixed retention. Given the study limitations, fixed retention should be considered with caution in restricting tooth overeruption in unopposed molars.

## Background

A plethora of terms including overeruption [1], hypereruption [2], supraeruption [3], supereruption [4], and continuous eruption [5, 6] have been used to describe the tendency of tooth movement in an occlusal direction following loss of antagonist contact. This phenomenon has been claimed to induce occlusal interferences and changes in the dental equilibrium [2, 7]. A 12-year study in females with missing opposed and/or adjacent molars showed 4.9 times higher risk of overeruption of ≥2 mm in unopposed molars [8]. Not all teeth without antagonist will necessarily overerupt, not even in a long-term perspective. Examination of the position of molars that had been unopposed for a long period showed that 18 % of the teeth exhibited no signs of overeruption [1]. Maxillary unopposed teeth appear to migrate vertically more than mandibular [4, 8] with the eruption being most pronounced during the first years after the loss of the opposed tooth [9]. Age and periodontal condition may be associated with the severity of changes. A higher incidence of severe overeruption has been observed in studies with younger age and periodontally affected groups [10]. Unlike young age [11], compromised periodontal condition was not associated with the severity of changes in animal experiments [12]. A recent systematic review on the treatment need for posterior bounded edentulous spaces [10] demonstrated that overeruption was limited to 2 mm for most studies reviewed. However, the authors classified the quality of evidence as very low and concluded that tooth replacement should not be considered as the mainstay of therapy.

Placement of etched metal splints on the lingual surfaces of unopposed molars has been recommended to counteract tooth extrusion [13, 14]. According to the retention protocol of a Class II Division 1 malocclusion treatment technique involving extraction of maxillary first molars, multistranded sectional wires are bonded on mandibular first and second molars to prevent vertical displacement of the out-of-occlusion second molars as a result of the late eruption of maxillary third molars [15, 16]. To the authors’ knowledge, no clinical study has been published so far aiming to explore the potential overeruption of nonoccluding teeth retained with sectional wires.

The objective of this study was to investigate whether overeruption occurred in unopposed mandibular second molars with multistranded fixed retainers in patients treated with orthodontic extraction of maxillary first molars.

## Methods

A total of 65 consecutively treated Class II Division 1 cases (28 females, 37 males) were retrieved from the archives of a private practice. They comprised a subgroup from a prospective clinical study [15] with the following inclusion criteria: Caucasian Whites, overjet ≥4 mm, no missing tooth or agenesis including maxillary third molars, permanent dentition, available panoramic radiographs after treatment (T1) and at a follow-up (T2) and treatment with 2-maxillary first molar extraction and Begg fixed appliances. The treatment approach has been described in detail in the literature [15, 16]. In case that the mandibular second molar had not occluded with the antagonists at the time of appliance removal, 0.195-in. buccal retention wires (Wildcat, GAC, Central Islip, NY, US) were placed on the mandibular first and second molars to inhibit unwanted vertical tooth movement of the teeth without occlusal contacts. Based on the presence of bonded buccal retainers on the mandibular first and second molars at two posttreatment timepoints (T1, T2), the subjects were allocated to the study-retention group (12 females, 18 males; mean age at T1, 15.2 years; SD, 1.6 years), and the control-nonretention group (16 females, 19 males; mean age at T1, 16.2 years; SD, 1.7 years) (Table 1).

**Table 1:** Summary statistics (means, SD in parentheses) of the retention and non-retention groups

	Retention group (n=30)	Non-retention group (n=35)
Males	18	19
Females	12	16
Age at T1 (years)	15.2 (1.6)	16.2 (1.7)
Age at T2 (years)	17.6 (1.7)	18.6 (2.0)
T2-T1 interval (years)	2.4 (0.8)	2.4 (0.9)

**Fig. 1:**
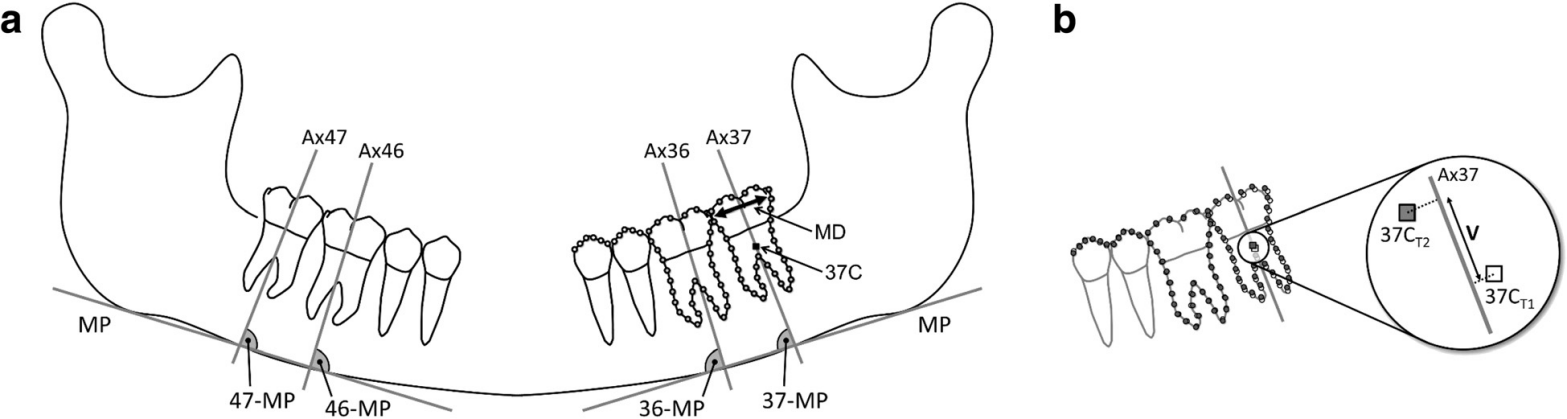
**a** Reference points and planes: mandibular plane (MP); Ax36, Ax37, Ax46, and Ax47: first and second molar long axes constructed by the midpoints of the occlusal surfaces and root apexes of the molars; mesiodistal dimension of second molar crown (MD); centroid of the mandibular second molar (37C); molar inclination angles: 36-MP, 37-MP, 46-MP, and 47-MP. **b** Best fit superimposition of panoramic radiographs taken at T1, T2; in *squares*: centroids of mandibular second molar at T1, T2 (37C_T1,_ 37C_T2_); movement of centroids along the molar long axis (V); *white circles*: digitization points at T1; *grey circles*: digitization points at T2

All panoramic radiographs were scanned (Epson Expression 1680 Pro, Suwa, Nagano, Japan; resolution of 600 dpi) and traced by the first author using a cephalometric analysis software (Viewbox 3.0; dHAL Software, Kifissia, Greece). The centroids of the mandibular right and left second molars were selected to represent the molar teeth. A set of 77 points lying on the outline of the teeth were digitized, 11 points on the occlusal surface of premolars and 33 points on each molar, 11 points on the mesial outline, 11 points on the distal outline, 4 points on the occlusal surface, and 7 points between the molar roots (Fig. 1a). The centroid of a collection of points is the average of the points, and its location is calculated by taking the average of the *x* and *y* coordinates of the points. In this study, the centroid of the second molar was computed as the average of outline points and subsequently transferred from the T2 to the T1 dataset by means of Procrustes and best fit superimpositions. By applying the first superimposition on the two molars and the occlusal surfaces of the two premolars of the buccal segment under investigation, the size between the two panoramic radiographs was adjusted. The second superimposition was carried out on the first molar and the occlusal surfaces of the premolars to measure the distance between the second molar centroids along the direction of the long axis of the tooth (distance V in Fig. 1b). Given the limitations of panoramic radiography in providing absolute linear measurements [17], we decided to express the molar movement as a percentage of its mesiodistal size. Therefore, the software was set to calculate the ratio of this distance (*V*) to the mesiodistal dimension of the mandibular second molar crown (MD), providing a percentage value for the occurring molar movement between T1 and T2; 37 V/MD, 47 V/MD. Assuming an average molar width value of 11 mm, 1 % of tooth movement corresponds to 0.11 mm. Molar inclination was determined in relation to the mandibular plane (MP) by the angles between the molar long axes and MP; 36-MP, 37-MP, 46-MP, 47-MP (Fig. 1a).

### Statistical analysis

Data analysis was carried out using a statistical software package (version 2.7.2; StatsDirect, Cheshire, UK). The measurements were tested for normality of distribution and equality of variance (F test). If the *F* test was significant, nonparametric alternatives (Mann-Whitney *U* and Wilcoxon signed-rank tests) instead of parametric methods (paired and unpaired *t* tests) were applied for intergroup comparisons between T1 and T2. Statistical significance was set at 5 %. To estimate reproducibility of measurements, 25 randomly selected pairs of tracings were replicated by the same examiner 2 weeks after the first series of tracings [18].

## Results

Reproducibility was assessed using the method of Bland and Altman [19]. The mean difference values for the repeated 37 V/MD and 47 V/MD measurements were 0.19 ± 4.24 % (95 % CI, −8.12 to 8.51) and 0.92 ± 3.40 % (95 % CI, −7.58 to 5.75).

Descriptive statistics for 36-MP, 37-MP, 46-MP, 47-MP, 37 V/MD and 47 V/MD, are summarized in Table 2. Comparison of T1 molar inclination values showed no significant differences between the retention and nonretention groups (*P* > 0.05) (Table 2). The mandibular left molars with fixed retention appeared at T1 slightly more mesially inclined than nonretention controls. The contralateral molars were slightly more upright in the retention than in the nonretention group. These trends in molar inclination persisted at T2 without reaching statistical significance (*P* > 0.05).

**Table 2:** Means, SD in parentheses of the molar inclination angles and movement percentages at T1 and T2 and P values, 95% CI of intergroup differences (unpaired t-test): Ret, retention group; Non, non-retention group; *, Mann–Whitney U test

	T1			
Measurement	Ret (n=30)	Non (n=35)	P value	95% CI
36-MP (°)	90.8 (4.9)	92.2 (6.9)	0.34	−1.54 to 4.40
37-MP (°)	91.1 (6.1)	91.2 (7.9)	0.97	−3.49 to 3.62
46-MP (°)	89.4 (5.2)	86.9 (5.5)	0.07	−5.11 to 0.22
47-MP (°)	89.0 (7.4)	85.7 (10.0)	0.13	−7.77 to 1.06
	T2			
Measurement	Ret (n=30)	Non (n=35)	P value	95% CI
36-MP (°)*	89.9 (4.4)	91.9 (7.1)	0.09	−1.00 to 4.98
37-MP (°)*	90.7 (4.8)	91.4 (7.2)	0.62	−2.38 to 3.76
46-MP (°)	88.3 (6.5)	86.7 (7.2)	0.32	−4.72 to 1.56
47-MP (°)	87.5 (7.2)	86.1 (10.1)	0.53	−5.80 to 2.99

No significant differences were found between T1 and T2 for either molar inclination angles or movement percentages (*P* > 0.05) (Table 3). Retained molars exhibited slightly increased mesial inclination whereas no clear patterns could be seen in the axial inclination changes of the counterparts without retention wires. On average, all molars overerupted during the observation period with this tendency being more prominent though not statistically significant in the nonretention molars.

**Table 3:** Means, SD in parentheses of the molar inclination angles and movement percentages between T1 and T2, and P values, 95% CI of intragroup differences (paired t-test): Ret, retention group; Non, non-retention group; *, *Wilcoxon* signed-rank test

	T2-T1					
Measurement	Ret (n=30)	P value	95% CI	Non (n=35)	P value	95% CI
36-MP (°)*	−0.9 (3.6)	0.18	−0.45 to 2.24	−0.3 (3.5)	0.58	−0.88 to 1.55
37-MP (°)*	−0.4 (4.4)	0.60	−1.21 to 2.05	0.2 (3.6)	0.73	−1.44 to 1.02
46-MP (°)	−1.1 (4.1)	0.16	−0.46 to 2.60	−0.2 (4.3)	0.77	−1.26 to 1.67
47-MP (°)	−1.5 (4.6)	0.09	−0.25 to 3.22	0.5 (5.2)	0.59	−2.27 to 1.32
37 V/MD (%)	1.0 (4.4)	0.23	−0.66 to 2.65	1.2 (5.2)	0.19	−0.60 to 2.98
47 V/MD (%)	0.5 (5.5)	0.61	−1.54 to 2.57	1.1 (5.7)	0.26	−0.84 to 3.06

## Discussion

A common belief among dental professionals is that molars without antagonists tend to overerupt leading to dental problems in the long-term perspective. A questionnaire survey among dentists on the perception of potential risks for molars without antagonists revealed that 85 % of the respondents believed that overeruption of the nonoccluding molars would occur. Interestingly, more than half of the dentists considered necessary to perform prosthodontics in the opposing arch to fill the edentulous space [20]. The influence of one-arch orthodontic extractions on the position of antagonists has been scarcely investigated in the past. Smith [21] observed that the distal aspect of the mandibular second molars overerupted significantly in subjects orthodontically treated with extraction of maxillary second molars compared to nonextraction controls. Crown tilting was likely to occur if partial occlusal contact had been established mesially with the distal portion of the occlusal surface of the opposing first molar.

Our study demonstrated statistically nonsignifant changes in molar positions determined by the mandibular plane and the movement of molar centroid along the tooth long axis regardless of whether sectional bonded retainers had been used or not. On average, slightly lower but not statistically significant overeruption rates were observed for the molars in the retention group compared to the control molars. Analyzing the results, the overeruption percentages between T1 and T2 ranged between 0.5–1.0 % and 1.1–1.2 % in the retention and nonretention mandibular second molars, which are translated into clinically insignificant changes of a tenth of millimetre.

Strictly speaking in clinical terms, the multistranded retention wires on mandibular first and second molars restrained the eruptive movement of unopposed second molars. Stated differently, the partial tooth contact with the antagonists in the control group appeared to be as efficient in preventing the general tendency for eruptive movement as the application of fixed retention in the opposing segment. In contrast to these findings, previous research has suggested that maintenance of vertical tooth position should not be clinically relied on partial tooth contact. In particular, Craddock found that teeth with partial tooth contact of 30 % or less occlusal overlap displayed a similar degree of overeruption to those without occlusal contact in adults missing teeth for over 5 years [22].

This study presents certain shortcomings, mainly related to the retrospective nature and the measurement method. No sample size calculation was performed prior to initiation of the study. All subjects with eligible radiographic records were included instead. Study cast measurements could have supplemented our radiographic methods to determine the overeruption rates. However, the lack of complete documentation made this option not feasible. On the other hand, model casting, i.e. impression and settling of casts may hide potentially errors, and such likelihood should not be underestimated [23]. The inclusion of dental casts might have been more favourable in case of upper arch measurements where the palatal rugae could serve as reliable landmarks for longitudinal cast analysis [24, 25]. Regarding the use of panoramic analysis, accuracy in overeruption and molar inclination measurements of the study might have been jeopardized by the inherent panoramic image distortions [26-28]. Registration of the relative vertical position of out-of-occlusion teeth on the panoramic radiographs was based on the assumption that the adjacent teeth had not moved during the observation period. To strengthen the tracing technique, we defined a wide list of digitization points extending from the distal outline of the mandibular second molar to the occlusal surface of the mandibular first premolar. However, the probability of tooth movement in the surrounding teeth cannot be neglected and may have partly contributed to the negative values in the vertical displacement of mandibular second molars. Moreover, the resulting growth of molar roots between observations in younger subjects should be also considered when interpreting the results. Finally, mechanical deformation of the retention wires during T1-T2 induced by biting on hard food [29], especially due to the rather increased intermolar wire span, might have also been involved. On the basis of current evidence, placement of mulistranded retention wires, though appeared to restrict overeruption of unopposed molars, cannot be fully warranted.

## Conclusions

Our study concluded that significant changes in the eruptive movement of unopposed mandibular second molars bonded with fixed sectional retainers did not occur during the observation period compared to nonretention counterparts with partial contact with the antagonists. In light of these findings, the use of fixed retainers to prevent the general eruptive tendency of nonoccluding molars may be effective but should be approached with caution.
